# Border Malaria Associated with Multidrug Resistance on Thailand-Myanmar and Thailand-Cambodia Borders: Transmission Dynamic, Vulnerability, and Surveillance

**DOI:** 10.1155/2013/363417

**Published:** 2013-06-25

**Authors:** Adisak Bhumiratana, Apiradee Intarapuk, Prapa Sorosjinda-Nunthawarasilp, Pannamas Maneekan, Surachart Koyadun

**Affiliations:** ^1^Department of Parasitology and Entomology, Faculty of Public Health, Mahidol University, 420/1 Rajvithi Road, Rajthewee, Bangkok 10400, Thailand; ^2^Department of Clinic, Faculty of Veterinary Medicine, Mahanakorn University of Technology, 140 Cheum-Sampan Road, Nong-Chok, Bangkok 10530, Thailand; ^3^Department of Fundamentals of Public Health, Faculty of Public Health, Burapha University, Chonburi 20131, Thailand; ^4^Department of Tropical Hygiene, Faculty of Tropical Medicine, Mahidol University, Bangkok 10400, Thailand; ^5^Ministry of Public Health, Department of Disease Control, The 11th Regional Office of Disease Prevention and Control, Nakhon Si Thammarat 80000, Thailand

## Abstract

This systematic review elaborates the concepts and impacts of border malaria, particularly on the emergence and spread of *Plasmodium falciparum* and *Plasmodium vivax* multidrug resistance (MDR) malaria on Thailand-Myanmar and Thailand-Cambodia borders. Border malaria encompasses any complex epidemiological settings of forest-related and forest fringe-related malaria, both regularly occurring in certain transmission areas and manifesting a trend of increased incidence in transmission prone areas along these borders, as the result of interconnections of human settlements and movement activities, cross-border population migrations, ecological changes, vector population dynamics, and multidrug resistance. For regional and global perspectives, this review analyzes and synthesizes the rationales pertaining to transmission dynamics and the vulnerabilities of border malaria that constrain surveillance and control of the world's most MDR falciparum and vivax malaria on these chaotic borders.

## 1. Overview of Border Malaria on Thailand-Myanmar and Thailand-Cambodia Borders

**Figure 1:**
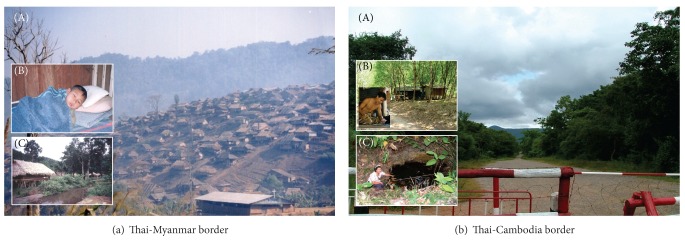
Complex epidemiological settings of border malaria. (a) A hilly 2202 km long Thai-Myanmar border where most investigations center on epidemiology, surveillance, and monitoring of MDR-associated BM in Tak province, Thailand, as one important area of studying MDR malaria in Southeast Asia region. (A) Numerous refugees revisiting either refugee camps temporarily or nearby pocket villages outside camps play important role in malaria transmission. (B) Schoolchildren including 12-year-old boy infected with *P. vivax* are at risk of malaria infections in endemic villages. (C) Breeding site of potent malaria vector, *Anopheles minimus*, is commonly found in endemic villages with irrigation and agricultural practices. (b) Similar to the endemic settings on or surrounding Thai-Myanmar border, a 798 km long Thai-Cambodia border where human settlements have extended to pocket villages with agricultural intensifications on plantations of rubber trees and fruit orchards. (A) Border crossings at immigration checkpoints are easier for migration of cross-border people due to geographical uplands, hills, hillside slope areas, and valleys. (B) Myanmar migrant workers including 32-year-old rubber plantation worker infected with *P. vivax* are at risk of malaria infections in endemic villages; both Myanmar and Cambodian migrant workers play key role in border malaria transmission. (C) Breeding site of potent *Anopheles dirus* and *Anopheles maculatus* is commonly close to human inhabitations with fruit orchards or rubber plantations.

The Greater Mekong Subregion (GMS), comprising the People's Republic of China (Yunnan PRC), Myanmar, Lao People's Democratic Republic (Lao PDR), Thailand, Cambodia, and Vietnam, has been taking into account the driving forces of population growth, urbanization, socioeconomy and globalization, and human/social development [1–4]. The revolutionary socioeconomy and globalization are among these plausible drivers that exert their interconnections through border trading, modern modes of domestic and intercountry transport, international travel, cross-border movement, and immigration of GMS people [1–6]. As consequences of these human activities, the GMS has continually faced broadly arrayed border health agendas by virtue of national health policy of each GMS country and, internationally, within the GMS countries in collaboration with enterprising counterparts [7–9]. Among border health concerns, malaria along the international borders becomes an important public health problem in the contemporary GMS countries despite the fact that strengthening capacity buildings of their public health systems and services has been extensively implemented nationwide [7–10]. Chronically, border malaria (BM) on Thailand-Myanmar and Thailand-Cambodia borders is a topic of regional and national public health concerns (Figure 1) [11]. This life-threatening disease is caused by *Anopheles* mosquito-borne transmission of two main human malaria parasites, *Plasmodium falciparum* and *Plasmodium vivax* [11]. Notably, the emergence and spread of multidrug-resistant (MDR) malaria, especially *P. falciparum* which is more recently resistant to artemisinins, appear to be unabated as they are underway of control through established systems, mechanisms, protocols, and response activities by the National Malaria Control Programs (NMCPs) in these affected countries, in collaboration and cooperation with the World Health Organization (WHO) and other concerted international partners [11–16]. Also, *P. vivax* MDR malaria becomes increasingly important because there has been a growing trend of increased incidence in certain transmission areas in the border areas, as it is appearing to be resistant to chloroquine [11–17].

Existing NMCPs, subsidized by the Global Fund Malaria (GFM) program, have been scaling up coverage and expansion services of global malaria control strategies, that is, rapid diagnosis and prompt treatment using rapid diagnostic tests (RDTs), and artemisinin-based combination therapies (ACTs), and other vector control using insecticide-treated nets (ITNs)/long-lasting insecticidal nets (LLINs) in a combination of indoor residual spraying (IRS), to be implemented in all geographically defined transmission control areas. As for Thailand, Thai NMCP has projected not only strengthening of surveillance and monitoring of MDR malaria but also coverage and expansion services of malaria control measures in malaria-endemic provinces along the border areas, as there is the exemption from the epidemics of MDR malaria [11–18]. Nonetheless, BM risk situations are likely due to its transmission dynamics underlying the geographical disparities and backgrounds of border people and the complexities of affected people and communities, local health sectors, and other stakeholders that all interplay in implementing those pragmatic strategies in different transmission control areas on or close to Thai-Myanmar and Thai-Cambodia borders. Regarding human settlements and intensive movements of the populations across the borders, we should gather needed data/information on the force of the circumstances that can bring about the complex BM and, concurrently, monitor the magnitude of BM transmission dynamics. Little is known about the complex BM or whether it is directly related to human activities other than ecological disturbances in developed and planned areas as a result of socioeconomic development and globalization. Occupational and behavioral risks for border people may result in increased susceptibility due to human-vector combinations relating to vector population adaptations and dynamics in transmission prone areas on the borders. Thus, it is essential for us to better understand what factors are the link to the complex BM settings and the vulnerability in how BM transmission dynamics can occur with the spread of MDR malaria. Furthermore, if there are limits of averting and monitoring MDR malaria in the GMS, the entanglement of BM will offer challenges to the achievement of progressive targets in the overall context of GMS human resource and health strategic development and the broader context of the eight Millennium Development Goals (MDGs) [19].

## 2. Border Malaria Epidemiology

### 2.1. Complex Border Malaria Settings and Dynamics

BM encompasses any complex epidemiological settings of forest-related and forest fringe-related malaria [11, 20, 21], both regularly occurring in certain transmission areas and possibly manifesting a trend of increased incidence in transmission prone areas along Thai-Myanmar (Figure 1(a)) or Thai-Cambodia border (Figure 1(b)), as a result of interconnections between human settlements and movement activities, border crossings, cross-border migrations, ecological changes, vector population dynamics, and multidrug resistance. The vulnerable population, especially whenever there are border crossings of local border people and cross-border migrations of migrant workers from Myanmar and Cambodia to Thailand, acquires naturally mosquito-borne malaria infection due either to risk behaviors, particularly regarded as those either improperly using ITNs/LLINs and other defensive measures or uncovered by household-level implementation of malaria control measures used in their NMCPs [11–18, 20–22], or to occupational exposures, particularly regarded as those involved in community or social services, crop plantations, forestry, mining, development projects, and tourism. 

**Figure 2:**
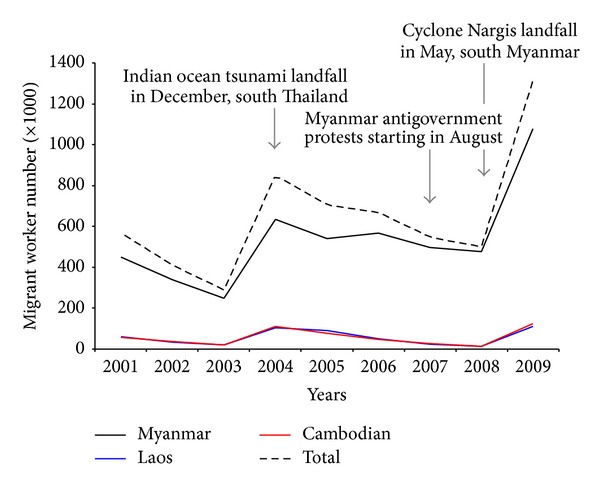
Trend of foreign migrant workers (Myanmars, Laos, and Cambodians) subject to the registration of work permits, according to provincial prorata demands in Thailand, 2001–2009. Regarded as the Section 13 of the Working of Alien Act, B.E. 2551 (2008), these cross-border migrant workers as illegal immigrants can apply for the engagement in officially permitted works as notified with regard to national security and social impacts in the government gazette by the Council of Ministers. Among foreign migrant workers, dynamic movements of Myanmar migrant workers are likely to be forced by some push effects. Data were modified from the Office of Foreign Workers Administration, Department of Employment, Ministry of Labor, Thailand (http://wp.doe.go.th/).

BM can affect all age groups but appears to be dynamic in human settlements and movement activities. Local border people living in the pocket villages alongside Thai-Myanmar and Thai-Cambodia borders include distinctively ethnic minority groups without nationality, especially on the Thai-Myanmar border. They all vary in socio-cultures, languages, and lifestyles. These border people of all ages are at risk of malaria infection if their hilly pocket villages situated in the forests on or near the borders are geographically associated with autochthonous malaria. Regarding the forest fringe villages or villages situated distant from the forests, adulthood rather than childhood infections are more prevalent due to workmen's forest activities such as logging, bamboo cutting, charcoaling, and foraging during revisiting and staying at the forests. Cross-border people, on the other hand, are at the greater risk of malaria infections because they frequently revisit forestlands, forest fringe areas, or forested plantations at multiple locations on or surrounding the border due either to border crossings or to cross-border migrations [23]. Border crossings can be defined as movement activities of local border people between the countries that occur with or without passing border control checkpoints of each land border. The movement activities are usually related to their normal lifestyles. Cross-border migrations can be defined as movement activities of cross-border people from a country origin to a country destination within these countries that occur when they cross the border with or without passing border control checkpoints of each land border. Their movement activities are normally described by “Push and Pull” effects or they can be grouped into external and internal migrations in that they intentionally migrate into a host country for either short-term or long-term immigration with different channels of migration [23–25]. Apart from an immigrant who migrates to another country usually for permanent residence, cross-border migrant workers (MWs) such as Myanmar migrant workers (MMWs), Cambodia migrant workers (CMWs), and Lao migrant workers (LMWs) usually migrate to Thailand (Figure 2) as they are subject to the registration of work permits based upon policy and domestic demands of labor forces in Thailand as a host country. Intensive movements of these substantial MWs are radically accelerated by economic driver, whereas movements of minority groups and refugees are largely driven by politics and human securities. Excluding both diverse minority groups and refugees residing on the borders, there is a substantial number of MMWs, as compared to CMWs and LMWs; accordingly, movements of these dynamic cross-border MWs reflect border health risk situations. More interestingly, it is thought that they play role in transmission dynamics of malaria and other transborder diseases while contributing to the entanglement of public health efforts between Thai-Myanmar and Thai-Cambodia borders.

### 2.2. Vulnerability of Border Malaria

**Figure 3:**
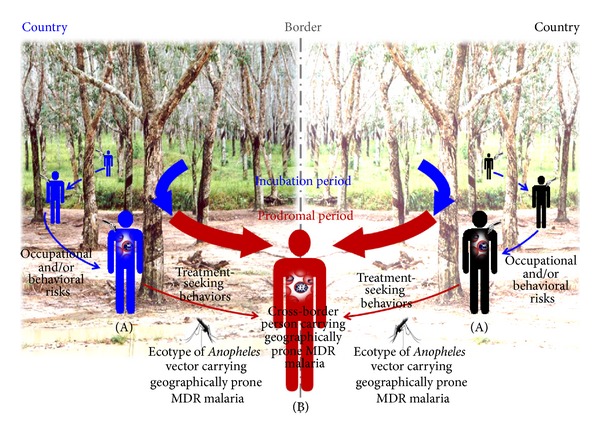
BM transmission dynamics and vulnerability. (A) Any cross-border person carrying malaria infection during an incubation period is exposed to multiple bites of *Anopheles* vectors at multiple locations on or close to the border due to occupational and/or behavioral risks and, vice versa, can spread malaria during a prodromal period until seeking treatment. (B) In MDR-associated BM setting, it is possible that any cross-border person carries geographically prone MDR malaria that can be epidemiologically linked to the ecotypes of *Anopheles* vectors carrying geographically prone MDR malaria in certain transmission areas on or close to the border.

As mentioned earlier, the border crossings of the vulnerable groups including MWs and local border persons are epidemiologically linked with transmission dynamics of BM; both *P. falciparum* and *P. vivax* are predominant agents affecting them in each side of land borders.  Figure 3 illustrates the vulnerability in how contagion of malaria causes BM transmission dynamics in the border areas. Any person who contracts malaria naturally acquires the infection through bite(s) of infective *Anopheles* vectors, which are common faunas of the natural forests and forested plantations close to the border. This circumstance explains why malaria-afflicted local border persons are associated with border crossings. Unless people have all the proper prevention behaviors, they are likely to be exposed to multiple bites of *Anopheles* vectors at multiple locations on or surrounding the border, in the same way that occurs in malaria-associated rubber plantations [20]. They can in turn spread malaria until they seek treatment in either of land border areas. Due to their occupational or behavioral risks, malaria-contracted cases may or may not be epidemiologically linked to time and location at which they both came into contact with infective bite(s) of *Anopheles* vectors and recognized onset malaria fever during prodromal infection before treatment-seeking behaviors are properly conducted. Such cross-border persons carrying malaria infections, which may or may not be geographically associated with transmission focus in any land border, are unlikely to be early diagnosed, that is, to delay treatment. Therefore, it is usually unreliable to gather these epidemiological data. Without the clarity of the following terms (i.e., settlements, movement activities, exposure(s) to bite(s) of *Anopheles* vectors, onset malaria fever during prodromal infection, and treatment-seeking behaviors of these malaria-afflicted persons), any epidemiological investigations may mislead the conclusions, whichever the infection that is carried by any infected person is linked to a transmission focus with the infection origin situated in his/her pocket village. Subsequently, there may be improper isolation sources for any country or geographic area when such these malaria persons are first diagnosed in any land border's settings. In general, a cross-border person with any malaria infection is classified as an imported case when diagnosed by a mobile unit, local malaria clinic, or local hospital in Thailand as well as in Myanmar and Cambodia. By contrast, cross-border people in Thailand side exclude both frontline workers who regularly work at forest protection checkpoints along forestland borders and other major group of vigilant soldiers and border patrol police officers who are regularly positioned at different checkpoints along land borders between the countries. However, both vulnerable groups who contract malaria are not classified as imported cases but likely as indigenous and introduced malaria cases. In regard to the guidelines of their NMCPs, those developing fever can be screened, on weekly basis, for blood examinations of malaria in each of the land border side. For example, many trained Thai soldiers can perform early diagnosis using RDT and prompt treatment with a recommended ACT (artesunate-mefloquine) at extremely remote bases or units along Thai-Myanmar and Thai-Cambodia borders.

As such, the following vulnerabilities influence transmission dynamics of BM on Thai-Myanmar and Thai-Cambodia borders:

- malaria-infected persons who cross the borders are likely to have treatment delay prior to visiting a local malaria clinic or a malaria post (i.e., a community-directed health service unit that radically provides community outreach for malaria blood examination using RDT and prompt treatment using a recommended ACT for an individual infected in the pocket of endemic villages remotely located in a transmission control area where targeted by the GFM-supported NMCP) for malaria diagnosis and treatment in any land border settings. Moreover, there are increased risks because they are lost to followups and render occupational exposures susceptible to spreading malaria unless their protective behaviors are properly conducted;
- some border people have improper health-seeking behaviors or self-medications, especially when treated with either counterfeit/substandard antimalarial drugs or monotherapies with antimalarial drugs, which become one of the principal causes of emergence of MDR falciparum and vivax malaria along the border areas [11, 13–16, 21, 22];
- surveillance and monitoring of *P. falciparum* and *P. vivax* MDR malaria will continually extend to certain transmission areas or hotspots of emergence and spread as geographically well-defined MDR malaria or suspected MDR malaria on these borders because there is a growing trend of decreased efficacies of ACTs and other antimalarials used in the NMCPs and, accordingly, more details are reviewed in this paper;
- there are limits of vector control as well as surveillance and monitoring of *Anopheles* vectors carrying MDR malaria parasites on the borders [11, 13–16, 21, 22]. If BM associated with the spread of MDR continues unbridled, there will be increasing public health challenges to either independently ongoing or joint implementation of their NMCPs, as existing control measures and methods are obsolete.

Moreover, the current situation of malaria on these borders seems to reflect the effectiveness of the implementation of the NMCPs although the overall reduction of malaria-associated mortality and morbidity has been achieved [11, 13–15]. In addition to what are guided by the GFM program, either independent or joint implementation of the NMCPs needs enhanced capacity buildings and knowledge management, otherwise BM will jeopardize both the management activities and desired outcomes of MDR surveillance and control that are parts of the NMCPs.  Table 1 shows that BM coexists with other potentially transborder diseases [26]. Interestingly, one-third of cross-border Myanmar health workers involved in community health or social services developed malaria infections when they traveled and visited the pockets of endemic villages in Myanmar land border. Movement activities are likely to be the link to the infections. In addition to what is observed in local border people alone, border movement activities of malaria-developing cross-border persons have the potential to transmit MDR malaria as it is epidemiologically linked through multiple bites of *Anopheles* vectors at multiple locations. This possibility needs to be investigated further. On the other hand, local border people with malaria infections are more vulnerable to coinfections with lymphatic filariasis or human immunodeficiency virus (HIV), as compared to those cross-border health workers. This also gives rise to figuring out the extent to which the magnitude and distribution of coinfections are determined [26, 27]. Still, all these figures point to major challenges for policy makers, health planners, public health professionals, and scientists to gather the needed data/information, share and leverage data/information required for GMS health strategic plans, monitor the magnitude of BM and other border health-related problems, and, in particular, better understand the interconnections of human settlements and movement activities, cross-border migrations, ecological changes, vector population dynamics, and multidrug resistance.

## 3. Surveillance and Monitoring of MDR Falciparum Malaria

For decades, BM epidemiology in the GMS has been linked with the emergence and spread of MDR falciparum malaria parasites. Particularly in certain transmission areas on the Thai-Cambodia border, *P. falciparum* does develop antimalarial drug-specific resistance against choloquine, sulfadoxine-pyrimethamine, and mefloquine [11, 17]. The Thai-Cambodia border has chronically been the hotspot or initial transmission focus of resistance to monotherapies with these antimalarial drugs used in the GMS and Southeast Asia region. Between the early 1960s and late 1970s, the emergence of *P. falciparum* resistant to chloroquine and sulfadoxine-pyrimethamine had been soon followed by the spread of MDR falciparum malaria across the GMS, Southeast Asia, South Asia, and, extensively, to Africa. This disastrous chronicle eventually rendered first-line treatment with chloroquine or sulfadoxine-pyrimethamine ineffective against falciparum malaria in most endemic countries; particularly in Africa, choloquine resistance has resulted in significantly increased malaria-associated mortality in children [28–31]. Between the late 1980s and early 1990s, mefloquine either alone or in combination with sulfadoxine-pyrimethamine became the first-line treatment of falciparum malaria. Mefloquine resistance developed in 1990 or 6 years after its introduction in the border areas. Due to high rate of recrudescence associated with mefloquine or mefloquine plus sulfadoxine-pyrimethamine therapy, the attendant risk of resistance development has resulted from increasing high failure rates of mefloquine, especially in certain transmission areas along Thai-Cambodia and Thai-Myanmar borders [32].

Artemisinin and its derivatives (e.g., dihydroartemisinin, arte-ether, artemether, and artesunate) that had been used on a limited scale in China in the 1980s were recommended for use as oral, parenteral, and suppository artemisinin-based monotherapies (AMT) [33]. In the 1990s, Cambodia and Vietnam initially implemented orally self-administered AMT using artesunate on a large scale as first-line treatment of uncomplicated falciparum malaria, whereas Thailand was one of the first countries outside China and Vietnam to continually register formulations of AMTs, which were restrictedly used in hospitals as oral artesunate, intramuscular artemether, intravenous artesunate, and artesunate rectocaps, respectively. All registered AMTs were recommended for use in treatment of *P. falciparum* severe malaria. Nonetheless, there were high rates of recrudescence associated with AMT in Vietnam and, subsequently, the artemisinin derivatives in combination of other antimalarial drugs were strongly recommended to replace the AMT [34]. After 1995, the ACT using artesunate-mefloquine became the effective treatment for uncomplicated falciparum malaria in these areas [32]. In Thailand, artesunate-mefloquine treatment (i.e., 25 mg/kg mefloquine over 2 days + 12 mg/kg artesunate over 3 days) was deemed necessary for reducing early resistance as well as MDR in patients with *P. falciparum* infection, especially in certain transmission areas with high-grade MDR falciparum malaria close to the Thai-Myanmar and Thai-Cambodia borders. Cambodia was the first GMS country to adopt ACT using artesunate-mefloquine as first-line treatment for MDR falciparum malaria nationwide in 2000 soon after launching the AMTs. Likewise, as part of its national drug policy 2002, Myanmar adopted the use of ACTs as first-line therapy for uncomplicated falciparum malaria, which include artesunate-mefloquine and artemether-lumefantrine. With regard to the Mekong Roll Back Malaria Initiative 1999, the overall reduction of malaria-associated mortality and morbidity in the GMS had been achieved during 1998–2005 [11, 17, 21]. However, more extensively sentinel surveillance and monitoring of MDR falciparum malaria in different epidemiologic settings of Cambodia, Myanmar, and Thailand have shown its propensity to continually develop early resistance to artesunate-mefloquine [11, 17, 35–44]. The evidence relies on standard *in vitro* drug sensitivity tests, *in vivo* susceptibility or therapeutic efficacy tests (TES) for 14 or 28 days (i.e., adequate clinical and parasitological response (ACPR) versus early treatment failure (ETF)/late treatment failure (LTF) rates), and molecular marker-based PCR methods (for differentiation between treatment failure associated with recrudescence and reinfection) carried out by the NMCPs, as well as independent investigations at different time periods.

### 3.1. Cambodia-Thailand Border Malaria

Between 2001 and 2008, sentinel surveillance and monitoring MDR falciparum malaria focused primarily on the sentinel sites of three certain transmission areas with intensive population movements: Pailin, Battambang, and Pursat Provinces, northwest-western part of Cambodia [11, 17, 35]. The ideal hotspots are situated close to Thai-Cambodia border provinces, Chanthaburi and Trat. The places are also known for the selection of *P. falciparum* MDR parasites under pressures of choloquine, sulfadoxine-pyremethamine, and mefloquine. Both Chanthaburi and Trat provinces reported high grade mefloquine resistance (R3), that is, efficacy rate of <50% with 750 mg mefloquine. The surveillance is based on annual monitoring of antimalarial drug efficacy carried out by the Cambodia NMCP whose the national drug policy recommended using 12 mg/kg artesunate over 3 days and 20 mg/kg mefloquine over 2 days (i.e., day 0, 4 mg/kg artesunate + 10 mg/kg mefloquine; day 1, 4 mg/kg artesunate + 10 mg/kg mefloquine; day 2, 4 mg/kg artesunate). Between 2001 and 2003, Pailin reported escalating treatment failure rates of artesunate-mefloquine (AM) treatment, showing 10% ETF and 14% LTF, whereas in Battambang high failure rates (13–33% ETF) of artemether-lumefantrine (AL) (i.e., 20 mg/kg artemether and 120 mg/kg lumefantrine over 3 days) were observed. A 2004 TES study in Pailin by Alker et al. [36] reported that AM treatment had 34% parasite clearance rate on day 3 and 10% on day 4. *P. falciparum* multidrug resistance gene 1 (*pfmdr1*) is a principal predictor for treatment failure with mefloquine [37, 38]. Increase in copy number of *pfmdr1* was significantly associated with recrudescence, and patients with increasing *pfmdr1* copy number (3 or more) were 8-fold greater risk of recrudescence than those harboring parasites with <3 copies. Clearly, Lim et al. [39] conducted three TES studies in Battambang: 2002 AL, 2003-2004 AL, and 2003-2004 AM, and one TES study using 2004 AM in Pursat. It was likely to show high failure rates: 71–86% ACPR and 13–29% LTF with AL; and 92% ACPR and 7-8% LTF with AM. The LTF patients harbored parasites with increased *pfmdr1* copy number 2.4-fold higher than the ACPR patients only in AM group but not AL group. There was a significant association between increased ≥3 *pfmdr1* copies and treatment failure in AM group (7.8 times) but not with treatment failure in AL group. As the result of *in vitro* drug sensitivity of the *P. falciparum* isolates from Battambang and Pursat which determined mean 50% inhibitory concentration (IC_50_) values using a log probit approximation, the parasites with increased *pfmdr1* copy number independently decreased susceptibility to chloroquine (IC_50_ = 175.6 nM), quinine (IC_50_ = 172.3 nM), mefloquine (IC_50_ = 50.3 nM), lumefantrine (IC_50_ = 44.1 nM), artemether (IC_50_ = 7.6 nM), and artesunate (IC_50_ = 2.1 nM), respectively. There was significant difference in decreased susceptibility to mefloquine, lumefantrine, and artesunate between the parasites with increased copies and single copy of *pfmdr1*. These findings might reflect a 2003–2005 *in vitro* drug sensitivity test of Cambodian isolates by Cambodia NMCP, showing a trend of decreased sensitivity of *P. falciparum* to chloroquine, quinine, mefloquine, and artesunate although a TES study using AM still reported above 97% ACPR with a 28-day follow-up [17]. Meanwhile, there is a marked decline in treatment efficacy of AM in Trat province by Thai NMCP; with this regard, 93% ACPR observed by a 1997–1999 TES study and 86% ACPR observed by a 2002–2006 TES study. 

Does *P. falciparum* develop early resistance to artemisinins in the early 2000s or 10 years after a preexisting mefloquine resistance in the early 1990s? In 2008, Rogers et al. [40] revealed that in Kampot province, southern Cambodia, AM treatment seemed losing its efficacy; they observed 11% parasite clearance rate on day 3. Treatment failure was associated with increased *pfmdr1* copy number and elevated mefloquine IC_50_ but not with artesunate IC_50_. Higher mean mefloquine IC_50_ (90 nM) and artesunate IC_50_ (1.7 nM) were observed in patients with recrudescence, whereas in those who recovered, mefloquine IC_50_ was 56 nM and artesunate IC_50_ was 1.2 nM. Recrudescence of *P. falciparum* was related to high parasitemia, longer time to parasite clearance, and increased *pfmdr1* copy number. More interestingly, one *P. falciparum* isolate from a patient with treatment failure showed decreased sensitivity to mefloquine (IC_50_ = 130 nM) and artesunate (IC_50_ = 6.7 nM). At the present time, continuing artesunate-mefloquine treatment failure, first seen in Pailin province [41, 42] and later in Pursat province [43], is therefore fundamental to reflect early artemisinin resistance of *P. falciparum* across the Cambodia-Thai border as it appears to reduce *in vivo* susceptibility to artesunate or other artemisinin derivatives [14–17, 35, 40–44]. Preexisting mefloquine resistance plays a key role in determining a genetic basis of the emergence of artemisinin resistance in certain transmission areas with continuations of artesunate-mefloquine treatment.

### 3.2. Myanmar-Thailand Border Malaria

*P. falciparum* rapidly developed resistance to mefloquine on the Myanmar-Thailand border in the late 1980s and later early resistance to artesunate-mefloquine after 1995. Among pioneer studies, Price et al. [37, 38] had investigated a molecular basis of mefloquine resistance using *P. falciparum* isolates of a baseline 1990–2002 dataset originated from uncomplicated malaria patients along the northwestern Thai-Myanmar border areas and successfully provided the proof that the parasites with increased *pfmdr1* copy number are associated with decreased sensitivity to mefloquine alone or AM, although the underlying molecular mechanism remains unclear. From a 1991–1994 dataset [37] that represented 13% high grade failure with mefloquine monotherapy, there was significant association between parasites with increased *pfmdr1* copy number and higher median IC_50_ values for quinine (IC_50_ = 556.8 ng/mL), mefloquine (IC_50_ = 64.3 ng/mL), halofantrine (IC_50_ = 13.2 ng/mL), artesunate (IC_50_ = 2.4 ng/mL), and dihydroartemisinin (IC_50_ = 1.8 ng/mL), respectively. Among these antimalarials tested for parasites with multiple *pfmdr1* copies, copy number was significantly correlated with IC_50_ to mefloquine only, but not parasite density, mixed *P. falciparum* and *P. vivax* infections, clonality of infection, age, sex, and recrudescence. In Cox regression models for analyses of the relationship between *pfmdr1* and responses to both treatment regimes, *pfmdr1* copy number was an important predictor for treatment failures; the population attributable risks of treatment failure associated with increased *pfmdr1* copy number were 63% with mefloquine monotherapy (25 mg/kg) by day 28 and 54% with AM treatment (25 mg/kg mefloquine + 12 mg/kg artesunate over 3 days) by day 42. From a 1995–1997 dataset [38] that presented IC_50_ for a subpopulation of genotyped isolates (both single-clone and multiclone infections), *P. falciparum* was independently susceptible to quinine (IC_50_ = 347 ng/mL), chloroquine (IC_50_ = 92.1 ng/mL), mefloquine (IC_50_ = 41.3 ng/mL), halofantrine (IC_50_ = 11 ng/mL), and artesunate (IC_50_ = 2 ng/mL). *P. falciparum* MDR parasites had degrees of resistance: 94% to chloroquine (>100 nM, 51.6 ng/mL), 91% to mefloquine (>20 nM, 8.30 ng/mL), 75% to halofantrine (>5 nM, 2.68 ng/mL), and 64% to quinine (>500 nM, 258.3 ng/mL). The* pfmdr1* copy number was not associated with the clonality of infection, recrudescence, age, and sex. However, parasites with increased *pfmdr1* copy number (≥2 copies) were significantly associated with increased IC_50_ values of mefloquine and artesunate, compared to those with single copy number. Such findings of early resistance to artesunate-mefloquine after 1995 still remained to be established.

In Myanmar NMCP, first-line AM treatment was recommended for uncomplicated falciparum malaria since 2002 to replace monotherapies with single-dose 15 mg/kg mefloquine and combined single-dose 25 mg/kg sulfadoxine + 1.25 mg/kg pyrimethamine. Between 2002 and 2008, Myanmar however changed its drug policies, using 3-day ACTs, which differed from that used in Cambodia-Thai border area [11, 17, 35]. First-line drug treatment was recommended as 12 mg/kg artesunate over 3 days and 25 mg/kg mefloquine over 2 days (i.e., day 0, 4 mg/kg artesunate + 15 mg/kg mefloquine; day 1, 4 mg/kg artesunate + 10 mg/kg mefloquine; and day 2, 4 mg/kg artesunate). This AM regime had been used in some certain transmission areas until 2005. Fixed dose of 3-day AL by body weight that was used nationwide during 2005–2008 was soon followed by 3-day coformulated ACTs (i.e., day 0, AL; day 1, dihydroartemisinin-piperaquine (DHAP); day 2, AM). Similar to the Cambodia NMCP, the Myanmar NMCP operated extensively sentinel surveillance and monitoring MDR falciparum malaria in some certain transmission areas close to the international borders with intense population movements, where selection pressures with choloquine, sulfadoxine-pyremethamine, and mefloquine for *P. falciparum* MDR parasites have been well established [11, 17, 35, 37, 38].

A 2002–2006 study by Myanmar NMCP showed that AM was losing its efficacy to 90% ACPR across the country, as similar to AL. The Myanmar NMCP also claimed that a declining treatment efficacy of ACTs was observed since 2004 in Kawthaung (Thanintharyi division), southeastern part of Myanmar. Kawthaung is known for intense movements of Myanmar migrant workers to neighboring Ranong province, southern Thailand [23, 24]. Efficacy rates of 3 ACT regimes between 2005 and 2006 were as: 100% and 91% ACPR with AM; 93% and 82% ACPR with artesunate-amodiaquine (AA); 98% and 92% ACPR with AL. A 2007 TES study using AL and AA regimens for treatment of uncomplicated falciparum malaria had been carried out in 4 sentinel sites: Rakhine (Myanmar-Bangladesh), Kachin (Myanmar-China), Karen and Mon (Myanmar-Thailand) states. All areas reported the same 97–100% ACPR with AL as well as AM. More interestingly, five LTF cases with AL included 2 Mon patients with PCR-corrected recrudescence. Three LTF cases with AA included the same one Kayin and Mon patient with PCR-corrected recrudescence. Similar to Kawthaung, Mon state is known as an origin of intense population movements of Myanmar migrant workers to Thailand as a host country [23, 24]. A 2009 TES study challenged AL versus DHAP in two sentinel sites, Shwe Kyin (Bago division) and Kawthaung. Shwe Kyin reported 98% ACPR with AL and 100% with DHAP. Parasite clearance time on day 3 showed 9.5% parasitemia patients with AL and 4.2% parasitemia patients with DHAP. Kawthaung reported 95% ACPR with AL as well as DHAP, whereas 10% parasitemia patients with artemether-lumefantrine and 29.6% parasitemia patients with DHAP. All ACTs had longer time to clear parasitemia pertaining to recrudescence. All the findings suggested that the emergence and spread of *P. falciparum* MDR malaria continue to evoke early artemisinin resistance across the country, particularly in some certain transmission areas close to Myanmar-Thai border. This emerging problem of early artemisinin resistance is likely to be supported by such evidence of AM resistance in 4 sentinel sites of Thai-Myanmar border provinces such as Mae Hong Son, Tak, Kanchanaburi, and Ranong [42]. Similar to Cambodia-Thai Chanthaburi and Trat border provinces, Tak had high-grade mefloquine resistance (R3) with 750 mg mefloquine. Both Mae Hong Son and Kanchanaburi provinces, with moderate grade resistance (R2), reported 50–70% efficacy rate. In Ranong, with low-grade resistance (R1), efficacy rate was >70%. Thai NMCP also claimed that dramatically decreased efficacy of AM was observed in Thai-Myanmar border provinces since 1997. Lately in 2009, it was clear to show that all the sentinel sites reported the same trend of decreased efficacy with PCR-corrected ACPR: 90% in Kanchanaburi, 87% in Mae Hong Son, 83% in Tak, and 80% in Ranong, respectively. AM treatment seemed to show longer parasite clearance time (percent of parasitemia patients on day 3): with 19% in Tak, 8% in Kanchanaburi, and 6% in Mae Hong Son; except 42% parasitemia on day 2 was observed in Ranong. Clearly, Aung-Pyae-Phyo et al. [45] demonstrated that this artemisinin resistance in *P. falciparum* emerged along the Thailand-Myanmar border at least 8 years ago based on a longitudinal study that measured the heritability (*H* ^2^) of parasite population with variation of emerging genotypes on parasite clearance rates by genotyping 96 single nucleotide polymorphisms across the *P. falciparum* genome in patients from western Thailand and western Cambodia. The variance of parasite clearance was compared within and between clonally identical parasites from more than two patients harboring either single-clone or multiple-clone infections. Patients with the same parasite genotypes had similar hours of parasite clearance half-life. The 2007–2010 dataset showed significantly stronger effects of parasite genotypes on parasite clearance half-life than that observed from 2001 to 2004; significantly increasing mean *H* ^2^ (0.7) in 2007–2010 adjusted by removal of 30% patients with the fastest clearing infections, compared to mean *H* ^2^ (0.3) in 2001–2004. This increase in *H* ^2^ was associated with emerging parasite genotypes that determined slow parasite clearance. Although a molecular marker for artemisinin resistance is unknown, the preexisting emergence and spread of *P. falciparum* MDR malaria on the Myanmar-Thailand border under selection pressures of quinolines, antifols, and sulfones strongly supported genetically determined artemisinin resistance *in P. falciparum*, as in the same way that evidence of resistance to artemisinins has been identified and confirmed on the Cambodia-Thailand border. Of note, *P. falciparum* MDR malaria in the GMS and South Asia appears to rethreaten public health systems more seriously than does globally prone chloroquine resistance [13–16, 35].

## 4. Surveillance and Monitoring of MDR Vivax Malaria

The NMCPs of the GMS countries including Cambodia, Myanmar, and Thailand recommended using chloroquine and primaquine as first-line treatments for *P. vivax*. Indeed, *P. vivax*, as known for which liver-stage hypnozoites cause relapse malaria, develops multidrug resistance more slowly in these countries than does *P. falciparum*. In many endemic countries, both chloroquine and primaquine are recommended as first-line treatments for *P. vivax*. Chloroquine acts as a blood schizontocide that kills asexual blood stages of *P. vivax* and *P. falciparum* and gametocytocide that kills *P. vivax* gametocytes but not those of *P. falciparum*. Primaquine acts as hypnozoitocide that kills liver-stage hypnozoites of *P. vivax* and *Plasmodium ovale* and tissue schizontocide that kills asexual liver stages and is potentially active as a blood schizontocide on *P. vivax*. In fact, the emergence of chloroquine-resistant *P. vivax* remains unclear; it may be associated with a relapse from the liver which reappears as parasitemia after chloroquine treatment with declining or impaired blood schizontocidal activity or it may result from a recrudescence which can recur as parasitemia; that is, the parasite originates from asexual blood-stage parasites that can manifest a survival after therapy.

Chloroquine resistance of *P. vivax* was first reported in 1989 in Papua New Guinea, and, in the early 1990s, Indonesia was facing the spread of chloroquine resistance across the country, leading to chloroquine treatment failure [46–48]. In the early 2000s, emerging chloroquine resistance in the area was soon followed by spread to Southeast Asia (Myanmar [49], and Vietnam in 1997–2000 [50]), India in 1995 [51] and extending to South Americas [52–55]. The NMCPs in the GMS began to establish *in vivo* susceptibility studies rather than *in vitro* drug sensitivity tests or molecular marker-based PCR methods because the overall increase in *P. vivax* incidence has been addressed across the GMS countries [17, 35]. It is believed that *P. vivax* MDR malaria exerts molecular mechanisms involved in chloroquine resistance in a way that occurs in *P. falciparum*. But why does *P. vivax* MDR malaria develop faster chloroquine resistance geographically associated with Myanmar but not Cambodia and Thailand? Little is known about the extent to which chloroquine resistance of *P. vivax* MDR malaria parasites in certain and suspected transmission areas in the GMS; that is, it needs to be warranted. 

Baseline information on *P. vivax* sensitivity or resistance to chloroquine over time periods on Myanmar-Thailand border has been disseminated since 1995. Chotivanich et al. [56] conducted *in vitro* drug sensitivity test of *P. vivax* chloroquine resistance using* P. vivax* isolates taken between 1996 and 2001 from Tak, northwestern Thailand. *P. vivax* was independently sensitive to artesunate (IC_50_ = 0.5 ng/mL), pyrimethamine (IC_50_ = 8 ng/mL), amodiaquine (IC_50_ = 14 ng/mL), and chloroquine (IC_50_ = 50 ng/mL) but not mefloquine (IC_50_ = 127 ng/mL), quinine (IC_50_ = 308 ng/mL), and sulfadoxine-pyrimethamine 80 : 1 (IC_50_ = 800/10 ng/mL), respectively. A standard treatment for *P. vivax* infection in Myanmar was 1500 mg chloroquine over 3 days immediately followed by 45 mg primaquine and then weekly for 8 consecutive weeks. After the first case report in 1993, this chloroquine regime was losing its efficacy in some *P. vivax*-infected patients who developed recurrent parasitemias on day 3 and 14; eventually, all were radically cured after a repeat treatment with 1500 mg chloroquine without relapse or recurrent infection [49]. Between 2002 and 2003, Guthmann et al. [57] reported chloroquine resistance in Dawei (Mon State), Myanmar, by conducting an *in vivo* susceptibility study using WHO standard treatment protocol for *P. vivax*: 10 mg/kg over the first 2 days and 5 mg/kg on day 3, for adult patients. A 34% recurrence of parasitemias was likely to have delayed parasite clearance times as was treatment failures with chloroquine. Two recurrences on day 14 that had whole blood concentration of chloroquine above 100 ng/mL demonstrated resistance to chloroquine. Clearly, chloroquine was losing its efficacy because 13% of *P. vivax* isolates taken from Myanmar-Thailand border between 2003 and 2006 demonstrated a resistance to chloroquine [58]. It was likely that the emergence and spread of chloroquine resistance of *P. vivax* MDR malaria parasites occurred in the early 2000s across the country. A 2005–2007 TES study by Myanmar NMCP reported that chloroquine was losing its efficacy (% ACPR), especially in some certain transmission areas with degrees of chloroquine resistance: Buthidaung 92% in 2005, Lashio 95% in 2006, and Mandalay 97% in 2007 [22]. Regarding *in vitro* test of *P. vivax *chloroquine resistance in Bhthidaung, chloroquine seemed to have less sensitivity of 82%. Meanwhile, a 2006 TES study by Thai NMCP reported high efficacy rate above 98% ACPR with chloroquine in Tak and Ranong border provinces. On the Cambodia-Thailand border, where intense *P. vivax* infection exists, either Cambodia or Thai NMCP reported that chloroquine remains highly effective against *P. vivax*; 97–99% ACPR in Pailin and Pursat, Cambodia during 2003–2005; and above 98% ACPR in Sakeaw and Trat border provinces of Thailand in 2006 [22]. In 2008, Pailin reported 95% ACPR with chloroquine, as there were three cases who reappeared with parasitemia on day 28 and after 42-day follow-up; it is unclear whether longer time to parasitemia clearance is associated with recurrence or recrudescence [35].

## 5. Perspectives

Of note, the regional malaria situation in the GMS including Cambodia, Myanmar, and Thailand has shown substantial improvement in surveillance and control toward progressive malaria elimination. However, there is a growing trend of artemisinin resistance of *P. falciparum* MDR malaria [41, 45, 59–61] and chloroquine resistance of *P. vivax* MDR malaria [49, 50, 57, 58], both occurring on the Thai-Cambodia and Thai-Myanmar borders. The MDR malaria parasite populations, under the selective pressures of quinolones, antifols, and sulfones/sulfonamides, will likely develop substantial resistance against almost all available antimalarials. If unabated, transmission of this MDR-associated BM will spread across the GMS and from Southeast Asia to South Asia. This extremity of *P. falciparum* and *P. vivax* MDR malaria parasites has been subject to extensive surveillance and containment in the GMS [11, 13–17, 35]. Effective surveillance and control of MDR malaria transmission rely on up-to-date, comprehensive, and validated data/information on MDR malaria and drug efficacy. However, it is also important to comprehend transmission foci of resistance and geospatial and temporal transmission patterns of MDR-associated BM in epidemiologically complex BM settings on these chaotic borders.

Of course, with respect to surveillance and monitoring of MDR malaria in suspected or certain transmission areas on or surrounding the borders, we do need more data/information provided by* in vitro* drug sensitivity tests, molecular markers, and pharmacokinetic studies as monitoring tools for MDR-associated BM. Trend data/information on sensitivity to chloroquine, sulfadoxine-pyrimethamine, mefloquine, and on molecular markers for the resistance to these drugs provide useful in epidemiological survey of MDR malaria and, accordingly, for assessing treatment failure in patients. In particular, artemisinin resistance in *P. falciparum* on both Cambodia-Thai and Myanmar-Thai borders is epidemiologically linked with the spread of multigenic MDR parasites to which multiple subpopulations determined by distinctive antimalarial resistance alleles responsible for genetic differentiation are related [59, 60]. In any complex BM settings in the GMS, the multigenic MDR parasites which inbreeding of resistant parasites and low transmission intensity are related to each other in an ecological niche are influenced by the positive selection of pressures [59–62], including the human host immunity, anopheline vector immunity, and physiochemical environments such as antimalarials and insecticides, as well as phylogenetic constraints over time periods. Perhaps these vulnerabilities have effects on high levels of haplotype homozygozity of multigenic MDR parasites, as well as can shape their genetic makeup of artemisinin-resistant and artemisinin-sensitive traits [60]. If the selective pressure increases the fitness of multigenic MDR parasites, more MDR descendants will increase the tolerance of the parasite population with decreasing sensitivity to the antimalarial drugs mentioned above. Increase in MDR parasite fitness affects the mutations in the parasite population, which can establish advantageous drug-resistant genotypes [59–62]. Without a balanced selection, an appearance of a parasite population bottleneck will eventually reduce the genetic variation in the population or the genetic diversity of the population geographically confined to a transmission focus or geographically prone to transmission foci. Such artemisinin-resistant subpopulations of *P. falciparum* in western Cambodia or on Cambodia-Thai border are more likely to be complex in pattern of population structure, as indistinguishable of geographical differentiation [60]. In this regard, BM on Thai-Myanmar and Thai-Cambodia borders are well known for the rapid establishment of MDR falciparum malaria parasite populations, precisely due to the pressures of quinolines, antifols, and sulfones/sulfonamides. The intensity of these selective pressures is considered a factor underlying the mutations of putative drug resistance genes of *P. falciparum*, for example, encoding chloroquine resistance transporter (*Pfcrt*), multidrug resistance pump (*Pfmdr 1*), dihydrofolate reductase (*Pfdhfr*), dihydropteroate synthetase (*Pfdhps*), calcium-dependent sarcoplasmic/endoplasmic reticulum ATPase (*Pfatpase6*), and GTP-cyclohydrolase (*Pfgch 1*) [59, 63], in a given multigenic MDR parasite population. This positive selection will eventually decrease the susceptibility of the MDR parasites to these drugs *in vitro* and *in vivo*. For artemisinin resistance in *P. falciparum*, it has recently been suggested that, using a *P. falciparum*-specific single nucleotide polymorphisms (SNPs) array, a selective sweep within a 35 kb selected region on chromosome 13 [59, 61] is associated with reduction in parasite clearance rates after treatment. Several candidate genes encode amniomethyltransferase (glycine cleavage complex), heat shock protein 70 (stress response/molecular chaperone), lipoate synthase (lipoic acid biosynthesis), krox-like protein (S-glutathionylation/redox regulation of protein function), and other putatively conserved proteins. Due to the existence of hypermutable parasites, continuous data/information of which parasite clearance half-life estimates or parasite clearance rates are indicative of artemisinin-resistant parasites during the ACTs [13] will help better understand what resistant genotype is emerging under the selective pressures over time periods in suspected or certain transmission foci on the borders in the GMS and Southeast Asia.

For regional and global perspectives, understanding genetic basis of multigenic MDR malaria parasites in the complex BM settings on Thai-Myanmar and Thai-Cambodia borders will be a radical step for public health professionals and researchers to guide standardized data formats and to explore genetic basis of globally prone MDR malaria parasites. For instance, if we do point to routine surveillance and monitoring of MDR falciparum malaria parasites in each land border, we could develop the rationale that artemisinin-resistant* P. falciparum* MDR malaria spreading in transmission areas close to Cambodia-Thailand border is linked to artemisinin resistance in transmission areas close to Myanmar-Thailand border. It is very interesting to note that more Myanmar migrant workers than Cambodian migrant workers are involved in rubber plantations and fruit orchards in transmission areas of Chanthaburi, Trat, and Sakaew, close to Thai-Cambodia border. Cross-border migrations of MMWs from Myanmar-Thailand to Cambodia-Thailand borders (Figure 1(b)) are more likely to contribute to the acceleration or exacerbation of MDR falciparum malaria transmission than the adaptation and migration of *Anopheles* vectors endogenous to the ecological habitats on or near the borders. Moreover, if ecological changes occur with the adaptation and migration of *Anopheles* vectors (e.g., *An. dirus*, *An. minimus*, *An. maculatus*, and *An. aconitus*) in the forest and forest fringe areas [20] on or surrounding the borders, there is a need to collect and leverage entomologic data/information on potentiating spread of MDR falciparum and vivax malaria parasites. With this regard, molecular marker-based PCR methods and sequencing of drug resistance genes are proven useful in epidemiological and entomological surveillance and monitoring of MDR falciparum and vivax malaria parasites [64, 65].

Because there are a large number of pocket villages geographically associated with malaria on the borders, malaria control activities require a renewal of fund raising and allocation for strengthening capacity building in surveillance and control for border malaria, as well as in human resource development and management applied to the GMS countries. Notably, policies for reducing drug pressures on border malaria can be addressed as significant strategy while maintaining vector surveillance and control along each land border in the GMS. Indeed, border malaria control scheme packages, along with health administration and finances, health information management, and universal access to health care services to border people, are neither operated by a single country nor within the GMS countries. Understanding and controlling transmission dynamics of MDR-associated BM require an integrated effective GMS malaria control program [11, 13–17]. Better effective control is dependent upon collective and shared management solutions to ensure the national treatment protocols for each GMS country that can manage border malaria challenges. Appropriately designed systems and mechanisms of health service delivery and operation research for local health authorities and other stakeholders require data sharing and communication between and within the GMS countries.

## Figures and Tables

**Table 1:** ^
a^Seroepidemiological data of coinfection of malaria with lymphatic filariasis (LF) and human immunodeficiency virus (HIV).

Vulnerable cross-border	LF^c^			HIV^d^		
Myanmar population^b^	Positive	Negative	Total	Positive	Negative	Total
Health worker						
TBF-positive^e^	1	22	23	0	23	23
TBF-negative	1	42	43	0	43	43
** **Total	2^**f**^	**64**	**66**	**0**	**66**	**66**

Non-health worker						
TBF-positive^e^	0	7	7	1	6	7
TBF-negative	2	12	14	2	12	14
** **Total	2^**f**^	**19**	**21**	3^**g**^	**18**	**21**

^a^Data modified from our previously published findings of serological diagnosis of plasma samples of  ^b^87 cross-border Myanmars: 66 health workers involved in community health or social services in remotely mountainous pocket villages in Myanmar but based in clinics at refugee camps in Tak-Mae Hong Son border provinces, northwest Thailand, and 21 nonhealth workers who developed malaria-like onset fever and visited clinics or local hospital in Tak and are local border people. All the samples were examined using ^c^circulating filarial antigen detection by commercially available Og4C3 ELISA specific for *Wuchereria bancrofti* and ^d^anti-HIV antibody-based ELISA specific for HIV type 1 and/or 2, as described in Bhumiratana et al. [26].

^e^Using standard Giemsa-stained thick blood films (TBF), positive blood samples included infections with either single or mixed falciparum and vivax malaria.

^f,g^Infected male adults aged ≤35 years old, as no reporting of coinfection with malaria, LF, and HIV.
